# Dynamic interactions with neuroblasts promote oligodendrocyte progenitor migration to injured cortex

**DOI:** 10.1016/j.isci.2026.117318

**Published:** 2026-08-24

**Authors:** Yuriko Sobu, Nodoka Ito, Edward William Ko Uy, Mayaka Hashimoto, Kazuya Kuboyama, Chihiro Akazawa, Shinsuke Shibata, Hirohide Takebayashi, Hideyuki Okano, Vicente Herranz-Pérez, Kazunobu Sawamoto, Naoko Kaneko

**Affiliations:** 1 Laboratory of Neuronal Regeneration, Graduate School of Brain Science, Doshisha University, Kyotanabe 610-0394, Japan; 2 Department of Developmental and Regenerative Neurobiology, Institute of Brain Science, Nagoya City University Graduate School of Medical Sciences, Nagoya 467-8601, Japan; 3 Intractable Disease Research Center, Juntendo University Graduate School of Medicine, Tokyo 113-8421, Japan; 4 Division of Microscopic Anatomy, Niigata University Graduate School of Medical and Dental Sciences, Niigata, Japan; 5 Center for Anatomical Studies, Graduate School of Medicine, Kyoto University, Yoshida-Konoe-cho, Sakyo-ku, Kyoto 606-8501, Japan; 6 Keio University Regenerative Medicine Research Center, Research Gate Building TONOMACHI 2-4F, 3-25-10 Tonomachi, Kawasaki-Ku, Kawasaki, Kanagawa 210-0821, Japan; 7 Department of Cell Biology, Functional Biology and Physical Anthropology, University of Valencia, Burjassot, Spain; 8 Division of Neural Development and Regeneration, National Institute of Physiological Sciences, Okazaki 444-8585, Japan

**Keywords:** oligodendrocyte progenitor cell, neonatal brain injury, ventricular-subventricular zone, remyelination, adherens junction, collective cell migration

## Abstract

Oligodendrocyte progenitor cells (OPCs) generated in the ventricular-subventricular zone (V-SVZ) migrate long distances to sites of brain injury to contribute to remyelination, but the mechanisms guiding their efficient recruitment remain unclear. Using a neonatal cortical injury model combined with live imaging and three-dimensional culture, we show that V-SVZ-derived OPCs migrate toward lesions by interacting with migrating neuroblasts that share the same route. Neuroblast contact significantly enhances OPC motility even in the absence of external scaffolds. High-resolution imaging reveals that these heterotypic interactions are associated with punctate adherens junctions that form and dissolve dynamically during migration. These findings uncover a previously unrecognized mechanism in which transient, dynamic adherens junctions support cooperative migration between neuronal and glial progenitors to facilitate efficient recruitment of OPCs to injured brain tissue.

## Highlights

• V-SVZ-derived newborn OPCs migrate toward the injured cortex via interactions with neuroblasts
• Direct contact with migrating neuroblasts enhances OPC migration
• Discontinuous, dynamically remodeled adherens junctions form between migrating cells

## Graphical abstract

**Figure:**
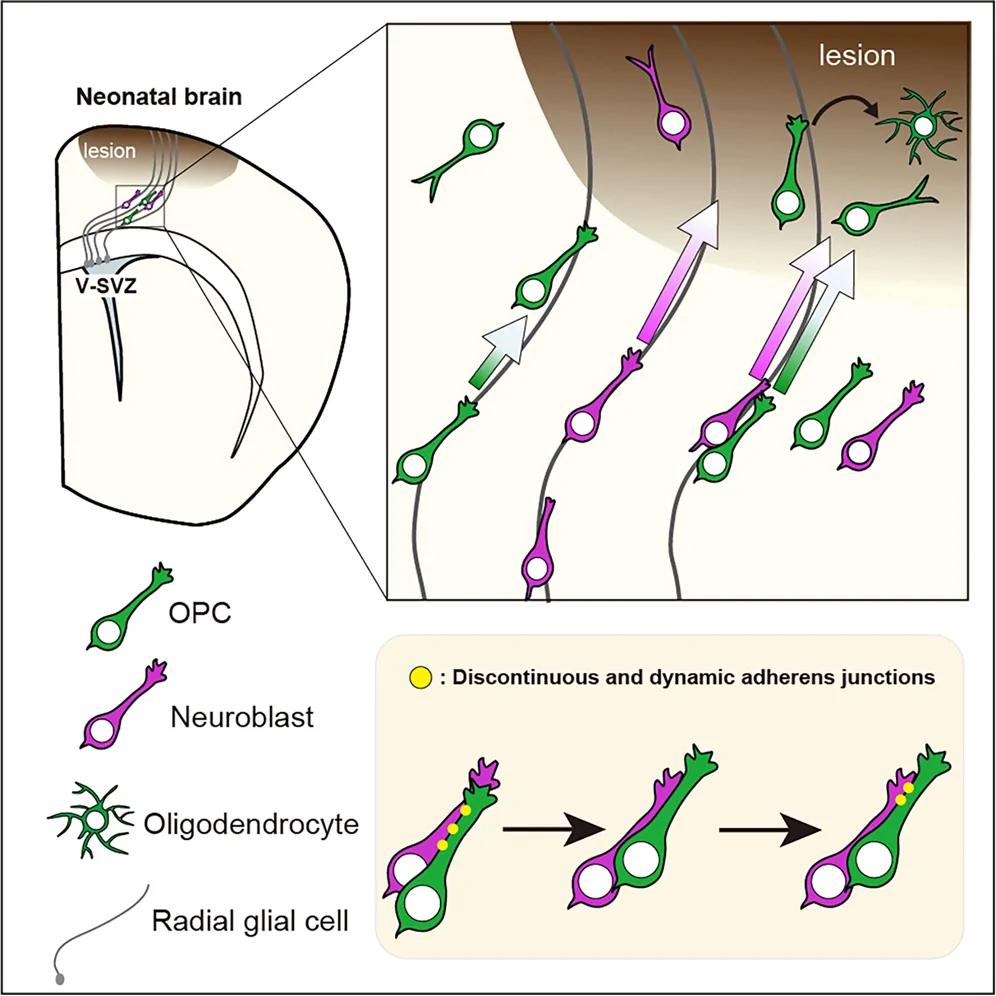

## Teaser

Biological sciences; Cell biology

## Introduction

Oligodendrocyte progenitor cells (OPCs) give rise to oligodendrocytes, which myelinate axons for saltatory conduction and provide metabolic support. In the postnatal brain, OPCs are widely distributed throughout the parenchyma,^1^^,^^2^ where they are referred to as parenchymal OPCs (pOPCs). In addition to this resident population, a subset of OPCs is generated from neural stem cells (NSCs) in the ventricular-subventricular zone (V-SVZ) along the walls of the lateral ventricles,^3^^,^^4^^,^^5^ although NSCs in this region primarily produce neurons. Under physiological conditions, newly generated immature neurons (neuroblasts) migrate along the rostral migratory stream (RMS) to the olfactory bulb, where they differentiate into interneurons,^6^^,^^7^ with only a minor and transient diversion toward the neocortex during the early postnatal period. V-SVZ-derived OPCs (V-SVZ-OPCs) migrate instead into adjacent forebrain regions, including the corpus callosum and striatum, where they differentiate into oligodendrocytes.^5^^,^^8^^,^^9^ Thus, the V-SVZ serves as a source of both neuroblasts and OPCs that migrate to distinct forebrain territories in the postnatal brain.

Under pathological conditions, injury-induced signals recruit subsets of V-SVZ-derived progenitors toward sites of tissue damage. While the majority of neuroblasts maintain their migration to the olfactory bulb, a fraction is redirected toward areas of neuronal loss, where they differentiate and contribute to functional recovery.^10^^,^^11^ Similarly, V-SVZ-OPCs can migrate toward damaged white matter and participate in remyelination following demyelinating insults, such as neonatal hypoxia-ischemia exposure and chemically induced white matter injury.^5^^,^^8^^,^^12^^,^^13^ Local pOPCs also proliferate robustly in response to demyelination and substantially contribute to remyelination.^14^^,^^15^ The relative contribution of V-SVZ-OPCs versus local pOPCs to remyelination, however, remains debated.^16^^,^^17^^,^^18^^,^^19^ Notably, accumulating evidence indicates that V-SVZ-OPCs possess enhanced migratory capacity, enabling them to traverse longer distances to reach deep-seated or distal lesions.^5^^,^^20^^,^^21^ While the mechanisms governing pOPC migration have been extensively characterized, particularly in demyelinating conditions,^15^ it remains unclear whether V-SVZ-derived OPCs utilize distinct migratory strategies or heterotypic interactions during their long-distance recruitment from the germinal niche to sites of injury in the postnatal brain.

Here, we investigated the migratory behavior of V-SVZ-OPCs in a neonatal mouse model of cortical injury. Live imaging of organotypic brain slices revealed that V-SVZ-OPCs migrate efficiently by directly interacting with neuroblasts. Using a three-dimensional culture system, we further identified dynamic adherens junctions (AJs) between migrating OPCs and neuroblasts. Together, our findings uncover a previously unrecognized interaction between neuronal and glial progenitors and suggest that interactions with neuroblasts facilitate OPC migration toward injured brain tissues.

## Results

### V-SVZ-derived bipolar-like OPCs migrate and differentiate into myelinating oligodendrocytes in the injured cortex

**Figure 1:**
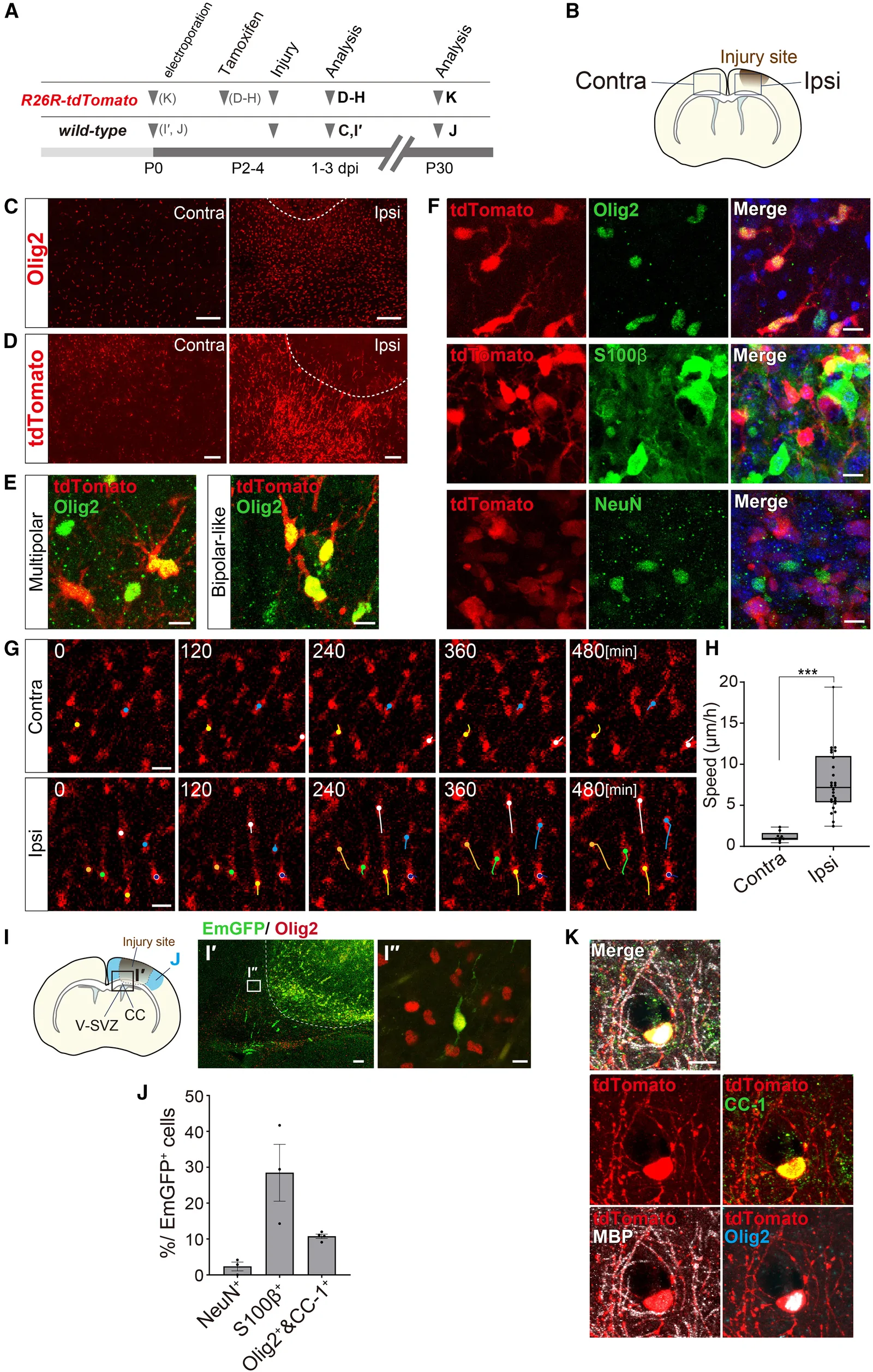
V-SVZ-derived OPCs migrate toward the injury site (A and B) Experimental protocol. The timeline (A) and schematic of a coronal brain section with the injury site (B) are shown. Dpi: days post-injury, Contra: contralateral, Ipsi: ipsilateral. (C–F) Coronal sections of the neocortices 2–3 days after injury. In wild-type mice, ipsilateral and contralateral neocortices were immunostained for Olig2 (C). In *Olig2-CreER;R26R-tdTomato* mice, low-magnification images of ipsilateral and contralateral neocortices (D), and higher magnification of representative images of multipolar- and bipolar-like tdTomato^+^Olig2^+^ cells (E) in the ipsilateral neocortices are shown. Images of tdTomato^+^ cells immunostained for the oligodendrocyte lineage marker Olig2, astrocyte marker S100β, and mature neuron marker NeuN (F). Cell nuclei were stained with Hoechst33342 (blue). The broken lines in (C) and (D) indicate the boundary of the lesion area. (G and H) Time-lapse imaging of tdTomato^+^ OPCs in the contralateral (top) and ipsilateral (bottom) neocortices of *Olig2-CreER;R26R-tdTomato* mice one day after lesion. Dots and lines indicate migration tracks of each bipolar-like migrating OPC over 480 min (G). The graph shows migration speeds of bipolar-like OPCs migrating toward the dorsal cortex in the ipsilateral and contralateral neocortices (H). Boxplots show the median, upper/lower quartiles (box), and min/max values (whiskers). A total of 26 ipsilateral cells and 9 contralateral cells from 2 independent animals were analyzed. ∗∗∗*p* < 0.001; Welch’s unpaired *t* test. (I and J) Fate mapping of fluorescent reporter-positive cells labeled by electroporation in ipsilateral neocortices in wild-type mice. Schematic of a coronal brain section showing the injury site, V-SVZ area, and corpus callosum (CC) in a wild-type mouse 2 days after injury (I). (I′) shows confocal images of the boxed region in (I) immunostained for Olig2 (red). A broken line indicates the boundary of the lesion area. (I″) shows a higher magnification image of the boxed region in (I′). The graph shows the proportion of labeled cells assigned to each differentiated cell type based on marker expression among labeled cells located within the ipsilateral neocortex (cyan area in I) at P30 (J). *n* = 4 animals for Olig2^+^/CC-1^+^, *n* = 3 animals for NeuN^+^ or S100β^+^, with 17–45 fluorescent reporter-positive cells per animal for each cell marker. Each dot represents one brain. Data are mean ± SEM. (K) R26R-tdTomato mice were electroporated with a Cre-expression vector into the V-SVZ at P0. Confocal images show tdTomato^+^ (red) cells migrated beyond CC in the ipsilateral neocortex at P30. Sections were immunostained for CC-1 (green), myelin protein MBP (gray), and Olig2 (cyan). Scale bars: 100 μm: (C), (D), and (I′); 10 μm: (E), (F), (I″), and (K); 20 μm: (G).

To investigate postnatal OPC migration toward cortical lesions, we induced a focal cryogenic injury in the neocortex at postnatal days (P) 2–4 (Figures 1A and 1B). This injury triggers the migration of V-SVZ-derived neuroblasts toward the lesion site within one week.^22^ Immunostaining for Olig2, a pan-lineage marker for oligodendrocytes, revealed a robust accumulation of Olig2-positive (Olig2^+^) cells within and surrounding the lesion core as early as 2 days post-injury (dpi) (Figure 1C). To further characterize OPC morphology and behavior, we utilized the *Olig2-CreER*^*TM*^*;Rosa26R-tdTomato* (*Olig2-CreER;R26R-tdTomato*) reporter line.^23^ In intact (contralateral) neocortex, tdTomato-expressing (tdTomato^+^) cells were almost evenly distributed, whereas in the lesioned hemisphere, as early as 2 dpi, they were densely distributed around the injury core (Figure 1D), consistent with the distribution of Olig2^+^ cells shown in Figure 1C. Morphologically, these cells fell into two categories: one population exhibited complex, multipolar processes with several branches, typical of pOPCs^24^ (Figures 1E and S1A). The second population extended a primary process toward the lesion center with or without a short process at the rear, suggesting active migration^25^ (Figure 1E, right). For simplicity, OPCs exhibiting a bipolar morphology with a leading process oriented toward the lesion were hereafter referred to as “bipolar-like” OPCs. In the neocortex of these mice, over 90% of tdTomato^+^ cells two days after tamoxifen administration co-expressed Olig2, but rarely expressed the astrocyte marker S100β or the mature neuron marker NeuN (Figure 1F), as previously reported in neonatal mice exposed to hypoxia/ischemia.^13^ CC-1 immunoreactivity, used as a marker of mature oligodendrocytes, was partially detected in tdTomato^+^ cells in the contralateral cortex but was rarely observed around the lesion (Figure S1B).

To directly observe dynamic behavior of the OPCs, we performed live imaging using acute brain slices from *Olig2-CreER;R26R-tdTomato* mice. The majority of tdTomato^+^ cells with multipolar morphology (presumably pOPCs) were stationary in both ipsilateral and contralateral cortices (Figure 1G and Video S1), which aligns with evidence that pOPCs become less migratory during postnatal development.^5^^,^^21^ Conversely, we identified a distinct subpopulation of bipolar-like tdTomato^+^ cells that exhibited robust, directed migration toward the lesion site (Figure 1G and Video S1). Such bipolar-like cells were rarely observed in the contralateral neocortex, and their basal migratory speed was significantly lower than that observed in the injured neocortex (Figure 1H), suggesting that this bipolar-like migratory phenotype is strongly induced by injury.

Video S1. OPC migration in the contralateral (left) and ipsilateral (right) hemispheres, related to Figure 1G Time-lapse imaging of tdTomato^+^ oligodendrocyte progenitor cells (OPCs) in the contra- (left) and ipsilateral (right) neocortices of *Olig2-CreER;R26R-tdTomato* mice one day after cortical injury. Trajectories of analyzed bipolar OPCs are shown by colored dots and lines. Representative multipolar OPCs are highlighted by gray rectangles, and their trajectories are also shown for comparison, although these cells were not included in the migration analysis. Images were acquired every 12 min. Scale bars: 20 μm.

To test whether migratory OPCs originated from the V-SVZ, we electroporated plasmids encoding fluorescent reporters into the V-SVZ at P0 in wild-type mice (Figure 1A). Two dpi, labeled cells were widely distributed along a trajectory extending from the V-SVZ toward the injured neocortex (Figure 1I and I′). While the majority were radial glial cells extending long processes toward the pial surface, we also identified bipolar-like EmGFP^+^ cells that co-expressed Olig2 near the lesion (Figure 1I″), morphologically resembling the migratory OPCs observed in live imaging (Figure 1G).

Four weeks after injury, we examined the long-term fate of V-SVZ-derived cells that had migrated into the ipsilateral neocortex (Figure S1C). While approximately 30% of labeled V-SVZ-derived cells expressed S100β as reported previously,^26^ more than 10% of labeled cells displayed the highly branched morphology typical of mature oligodendrocytes and were positive for both CC-1 and Olig2 (Figures 1J and S1D). In *Olig2-CreER;R26R-tdTomato* mice, EmGFP^+^ cells that were positive for CC-1 or S100β, but not a small population expressing NeuN, were labeled with tdTomato (Figure S1E), as expected given the glial-specific targeting of the *Olig2-CreER* line in this context, consistent with results from other injury models.^13^^,^^16^^,^^17^^,^^27^ Furthermore, using *R26R-tdTomato* mice in which V-SVZ cells were labeled by electroporation of a Cre-expression vector at P0, we found highly branched V-SVZ-derived cells that were positive for Olig2, CC-1, and the myelin marker MBP in the ipsilateral neocortex near the lesion (Figure 1K). Together, these data suggest that V-SVZ-derived bipolar-like OPCs actively migrate toward injury sites and contribute to the pool of mature myelinating oligodendrocytes.

### V-SVZ-derived OPCs utilize radial glial scaffolds for lesion-directed migration, paralleling neuroblast behavior

**Figure 2:**
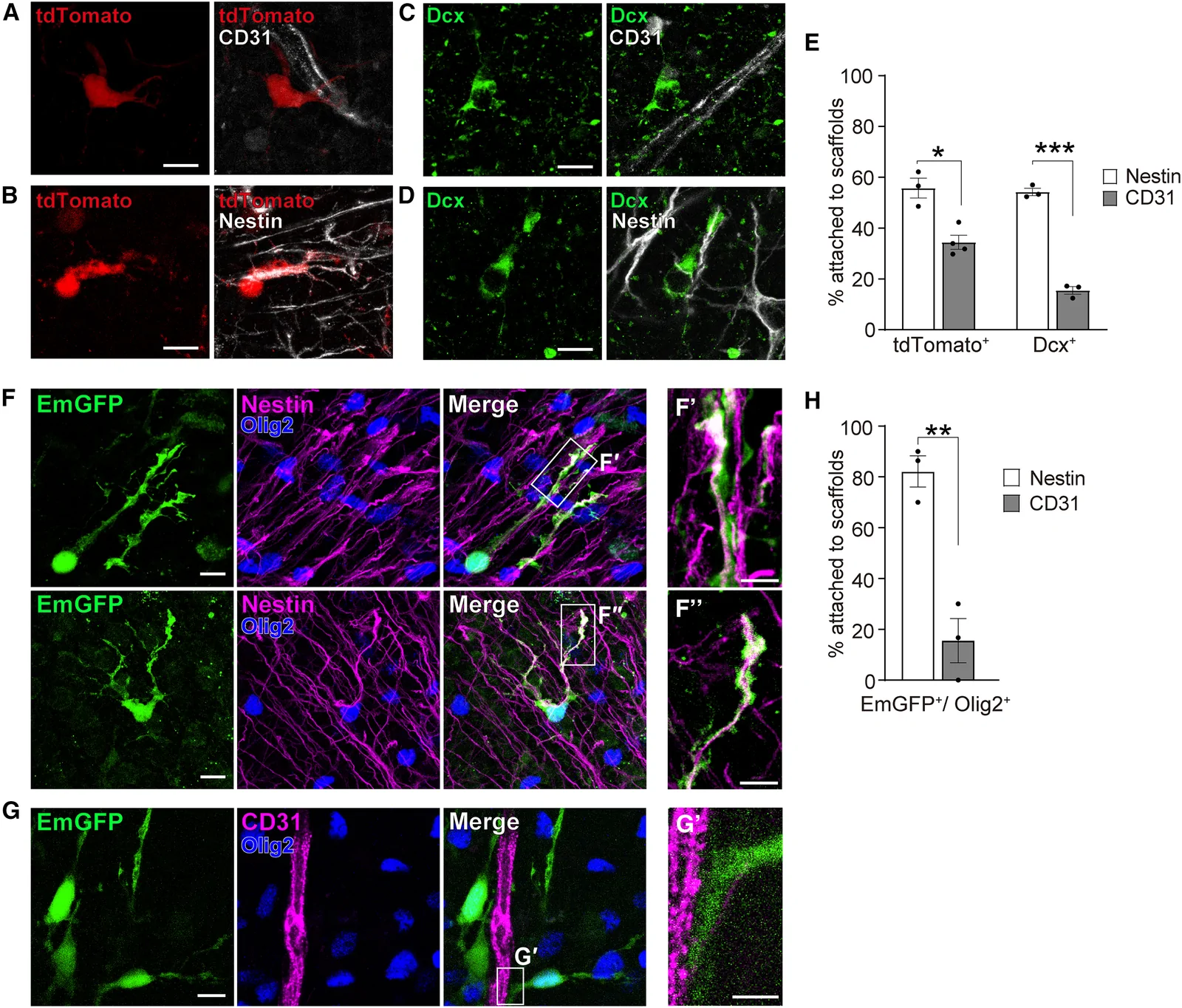
V-SVZ-derived OPCs utilize radial glial fibers as scaffolds rather than blood vessels (A–D) Coronal brain sections from postnatal day (P) 6 *Olig2-CreER;R26R-tdTomato* mice subjected to cortical lesion at P4, immunostained for the neuroblast marker Dcx (green) and either the radial glial marker Nestin (gray) or the endothelial marker CD31 (gray). Bipolar-like tdTomato^+^ oligodendrocyte progenitor cells (OPCs, red) (A and B) and Dcx^+^ neuroblasts (C and D) are shown in the injured neocortex. (E) Quantification of the percentages of tdTomato^+^ bipolar OPCs and Dcx^+^ neuroblasts associated with each scaffold type. Data are mean ± SEM. *n* = 4 animals for tdTomato^+^-CD31 and *n* = 3 animals for all other groups. Each dot represents one animal. ∗*p* < 0.05, ∗∗∗*p* < 0.001; Welch’s unpaired *t* test. (F and G) Coronal brain sections of P4 wild-type mice electroporated with an EmGFP expression vector at P0 and subjected to cortical lesion at P2, immunostained for the oligodendrocyte lineage marker Olig2 (blue) together with Nestin (magenta) or CD31 (magenta). EmGFP^+^Olig2^+^ cells (putative ventricular-subventricular zone-derived OPCs) are shown in the injured neocortex. (F′), (F″), and (G′) show higher magnification of the boxed region in (F) and (G). (H) Quantification of the percentage of EmGFP^+^Olig2^+^ cells associated with Nestin^+^ fibers or CD31^+^ blood vessels. Data are mean ± SEM. *n* = 3 animals. Each dot represents one animal. ∗∗*p* < 0.01; Welch’s unpaired *t* test. Scale bars: 10 μm: (A–D), (F), and (G); 1 μm: (F′), (F″), and (G′).

To elucidate the mechanisms of migration of V-SVZ-derived OPCs toward cortical lesions, we investigated the association of these cells with potential structural scaffolds. We evaluated three candidate scaffolds: (1) blood vessels, which guide embryonic OPCs^28^ and adult-born neuroblasts migrating toward the olfactory bulb^29^^,^^30^^,^^31^ and lesion areas,^32^^,^^33^ (2) axons, the ultimate targets for OPC-mediated myelination,^34^ and (3) radial glial fibers, which normally regress within a week after birth but are preserved following cortical injury, where they promote neuroblast migration.^22^ Brain sections from injured *Olig2-CreER;R26R-tdTomato* mice were immunostained for CD31 (vascular marker), neurofilament (axonal marker), and Nestin (radial glial marker), and the proportion of bipolar tdTomato^+^ OPCs in contact with each structure was quantified (Figures 2A–2E). Approximately 30% of OPCs contacted blood vessels; however, their entire processes were rarely aligned with the vasculature (Figures 2A and 2E). Despite the dense meshwork of neurofilament^+^ axons, OPCs showed minimal association with them (Figure S2). In contrast, more than 50% of bipolar-like OPCs were in direct contact with Nestin^+^ radial glial fibers, frequently extending their leading processes along these fibers (Figures 2B and 2E). The preference toward radial glia as a scaffold was similarly observed in Dcx^+^ cells of the same sample sets (Figures 2C–2E) and in V-SVZ-OPCs labeled by EmGFP electroporation in wild-type mice (Figures 2F–2H), consistent with our previous findings.^22^ These results indicate that radial glial fibers serve as a major migratory scaffold for both OPCs and neuroblasts during their journey from the V-SVZ to cortical lesions.

### Neuroblast interaction facilitates OPC migration toward the injured cortex

**Figure 3:**
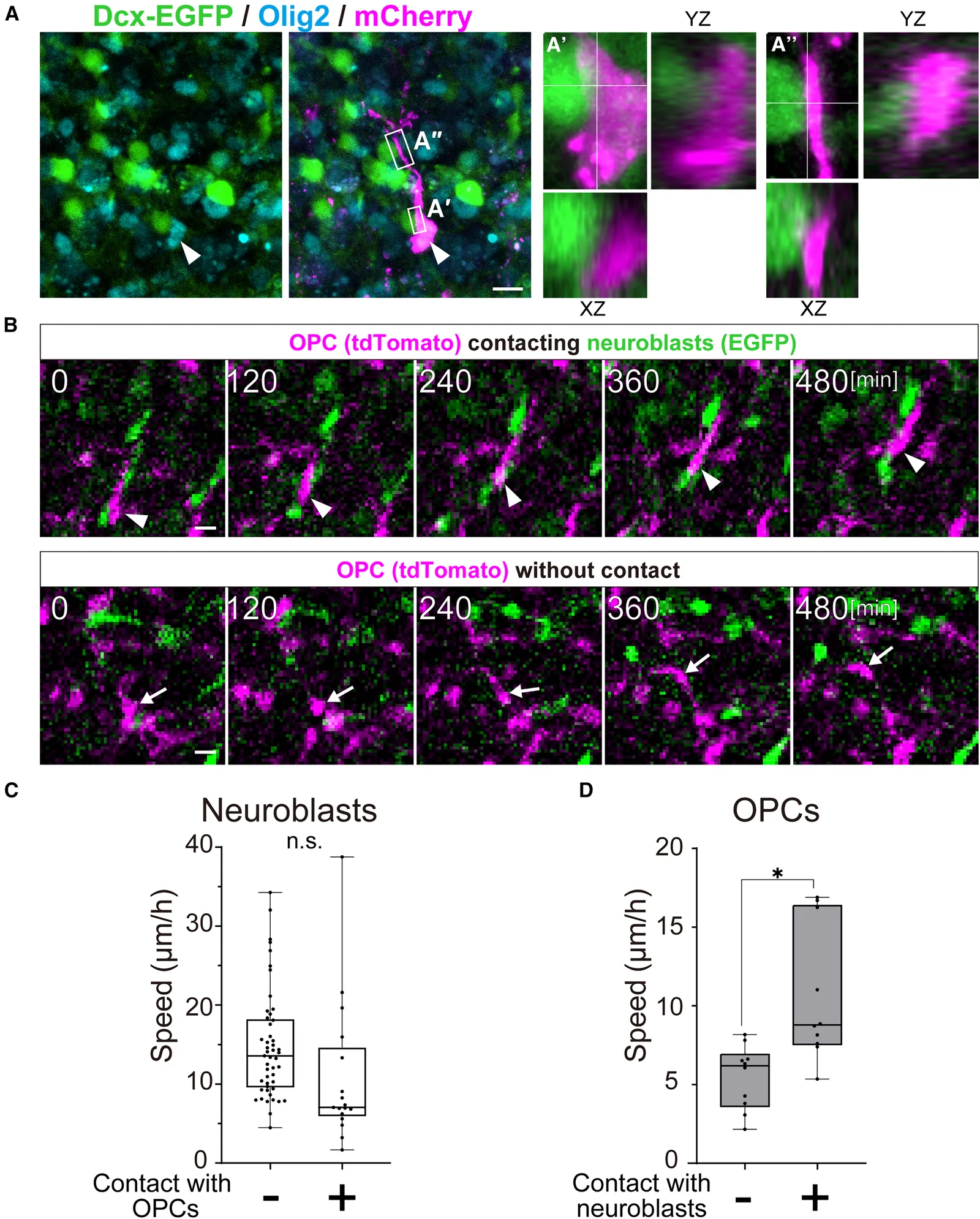
Contact with neuroblasts enhances OPC migration in the injured brain (A) Coronal brain section from a P4 *Dcx-EGFP* mouse electroporated with a pcDNA3.1-mCherry vector at P0 and subjected to cortical lesion at P2. An mCherry^+^Olig2^+^ oligodendrocyte progenitor cell (OPC, magenta/cyan) is shown in contact with EGFP^+^ neuroblasts (green) in the injured neocortex. (A′) and (A″) show higher magnification images and orthogonal views (YZ and XZ planes) of the boxed regions in (A). (B) Time-lapse imaging of P3 *Olig2-CreER;R26R-tdTomato;Dcx-EGFP* neocortices following cortical lesion at P2. tdTomato^+^ bipolar OPCs (magenta) migrating within the injured neocortex are shown either in contact with EGFP^+^ neuroblasts (top, arrowheads) or without contact (bottom, arrows). (C and D) Quantification of migration speeds toward the injured site. Neuroblast migration speed with and without OPC contact was compared between different cells (C). OPC migration speed with and without neuroblast contact in the same cells (D). Boxplots show median (center line), interquartile range (box), and min/max values (whiskers). Neuroblast: 19 contact cells and 62 non-contact cells from 2 independent experiments were analyzed. OPC: 11 cells exhibiting both contact and independent behaviors from 2 independent experiments were analyzed. Each dot represents the average speed of a cell. n.s.; Welch’s unpaired *t* test (C), ∗*p* < 0.05; paired *t* test (D). Scale bars: 10 μm: (A) and (B).

Given that V-SVZ-derived neuroblasts and OPCs share a migratory route, temporal window, and scaffold, we hypothesized that these cell types might interact extensively. To test this, the spatial relationship between V-SVZ-derived OPCs (mCherry^+^/Olig2^+^) and neuroblasts (EGFP^+^) was examined in mCherry-electroporated *Dcx-EGFP* mice.^35^ We occasionally observed close physical apposition between mCherry^+^ OPCs and EGFP^+^ neuroblasts (Figure 3A). To determine whether this contact influences migratory dynamics, we performed live imaging on acute brain slices from injured triple-transgenic mice (*Olig2-CreER;R26R-tdTomato*;*Dcx-EGFP*) (Figures 3B–3D). Mean migration speed of neuroblasts was higher than that of bipolar-like OPCs (13.8 ± 0.9 μm/h vs. 8.3 ± 0.9 μm/h, respectively). Notably, while neuroblast motility was not significantly altered by contact with OPCs (Figures 3B and 3C), OPCs exhibited a significant and transient increase in migration speed during periods of direct contact with neuroblasts (Figures 3B and 3D and Video S2), suggesting that neuroblasts can function as mobile cellular scaffolds that facilitate OPC migration. These findings suggest that heterotypic interactions with migrating neuroblasts promote cooperative migration and enhance the recruitment of OPCs toward the injury site.

Video S2. Migration of an OPC in contact with neuroblasts (left) and without contact (right) in the injured brain, related to Figure 3B Time-lapse imaging of P3 *Olig2-CreER;R26R-tdTomato;Dcx-EGFP* neocortical slices following cortical injury at P2. Oligodendrocyte progenitor cells (OPCs, magenta) migrating in contact with neuroblasts (green; left) or without neuroblast contact (right) are tracked with dots. Images were acquired every 12 min. Scale bars: 10 μm.

### Neuroblast contact promotes OPC migration independently of radial glial scaffolds

**Figure 4:**
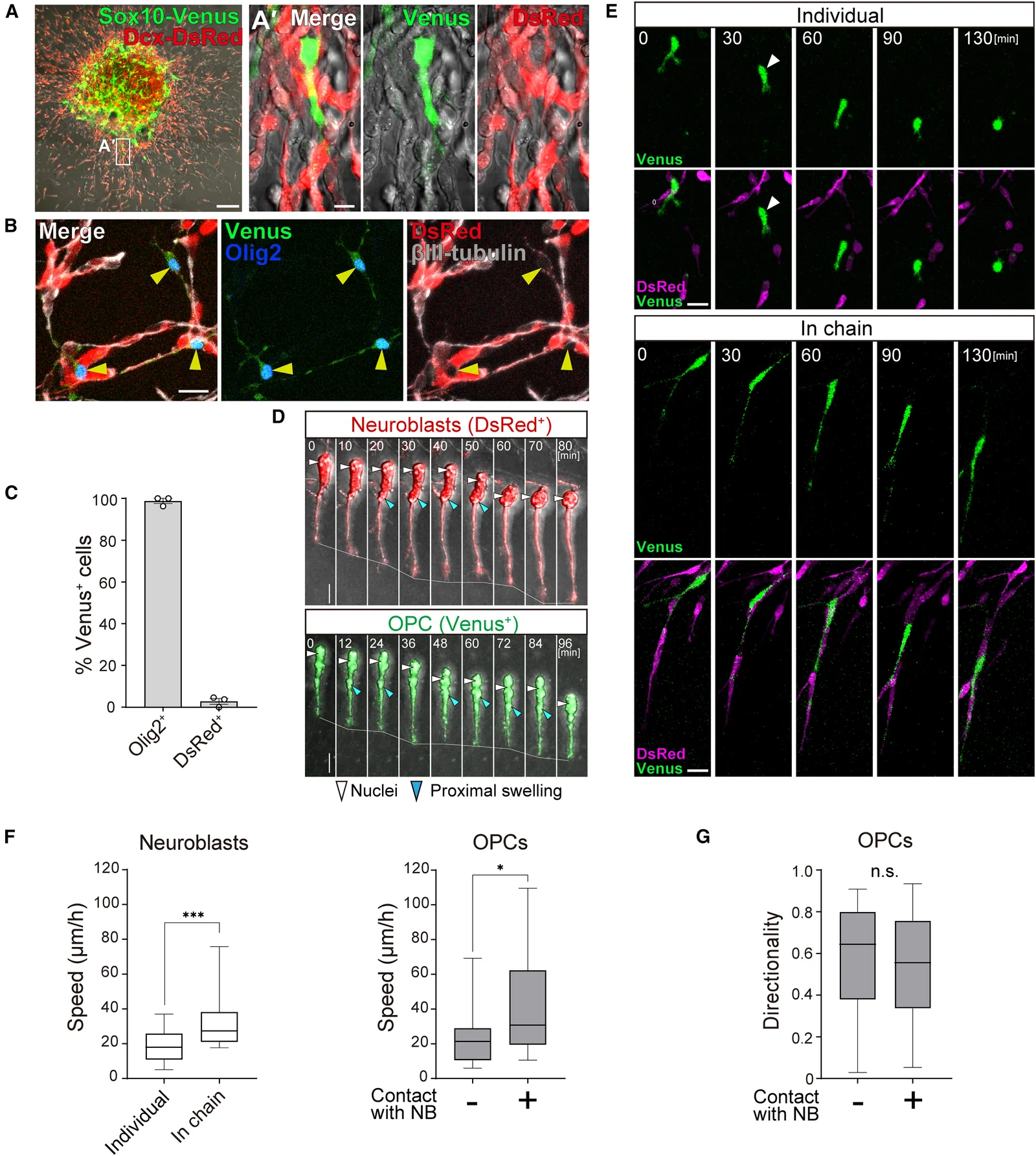
Neuroblast contact promotes scaffold-independent migration of OPCs *in vitro* (A) V-SVZ explants from *Sox10-Venus;Dcx-DsRed* neonatal mice embedded in Matrigel. Venus^+^ oligodendrocyte progenitors (OPCs) (green) and DsRed^+^ neuroblasts (red) are shown migrating radially from the explant. (A′) shows higher magnification of the boxed region in (A). (B) Immunostaining of explant cultures for Olig2 (blue) and βIII-tubulin (gray). (C) Quantification of the percentage of Venus^+^ cells co-expressing Olig2 or DsRed. Data are mean ± SEM. *n* = 3 independent cultures, including 14–27 Venus^+^ cells per culture. Each dot represents an independent experiment. (D) Representative saltatory migration of DsRed^+^ neuroblasts and Venus^+^ OPCs. White lines indicate leading process tips, white and blue arrowheads indicate nuclei and proximal swellings, respectively. (E) Time-lapse imaging of OPCs migrating individually (top) or within neuroblast chains (bottom). (F and G) Quantification of migration behaviors in explant cultures. Migration speed of neuroblasts (NBs) (F, left) and OPCs (F, right), and directionality index of OPCs (G) with or without incorporation into neuroblast chains are shown. Directionality index was calculated as net displacement divided by total migration path length. Boxplots show median (center line), interquartile range (box), and min/max values (whiskers). NBs in chains: 33 cells; individual NBs: 22 cells; OPCs with contact: 36 cells; OPCs without contact: 15 cells, from 5 independent cultures. ∗*p* < 0.05, ∗∗∗*p* < 0.001; Welch’s unpaired *t* test. Scale bars: 100 μm: (A); 10 μm: (A′) and (D); 20 μm: (B) and (E).

To investigate neuroblast-mediated enhancement of OPC migration in detail, we employed a three-dimensional culture system of V-SVZ explants from *Sox10-Venus* mice^36^ crossed with *Dcx-DsRed* mice^37^ (Figure 4A). In the V-SVZ of *Sox10-Venus* mice, nearly all Venus^+^ cells were positive for Olig2, but not for CC-1, a commonly used marker of mature oligodendrocytes, or for the neuronal marker βIII-tubulin (Figures 4B, 4C, and S3), indicating that OPCs were specifically labeled with Venus. Within two days of culture embedded in Matrigel, DsRed^+^ neuroblasts migrated outward frequently in chain-like clusters (Figure 4A). A much smaller population of Venus^+^ bipolar-like OPCs also migrated out of the explants, while many Venus^+^ cells with multipolar morphology remained inside the explant tissue.

Time-lapse imaging revealed that Venus^+^ bipolar-like OPCs exhibited a saltatory migration pattern—characterized by leading process extension, proximal cytoplasmic swelling, and subsequent somal translocation—resembling neuroblast behavior.^6^ We compared the migratory behavior of OPCs with or without neuroblast interaction (Figure 4E). Although migration speeds varied among cells, OPCs integrated into neuroblast chains migrated significantly faster than those moving alone, a phenomenon also observed in neuroblasts (Figure 4F). On the other hand, migration efficiency (defined as the ratio of net displacement to total track length) showed no significant difference between the two groups (Figure 4G). In addition, contact between migrating OPCs was not observed during the analysis (0 of 51 bipolar OPCs contacted another OPC). Together, these findings showed that neuroblast contact increases OPC motility (speed) without affecting directional persistence. These results indicate that direct contact with neuroblast chains is sufficient to enhance OPC motility, even in the absence of *in vivo* scaffolds such as radial glia or vasculature.

### Migrating OPC-neuroblast contact involves dynamic, punctate AJs

**Figure 5:**
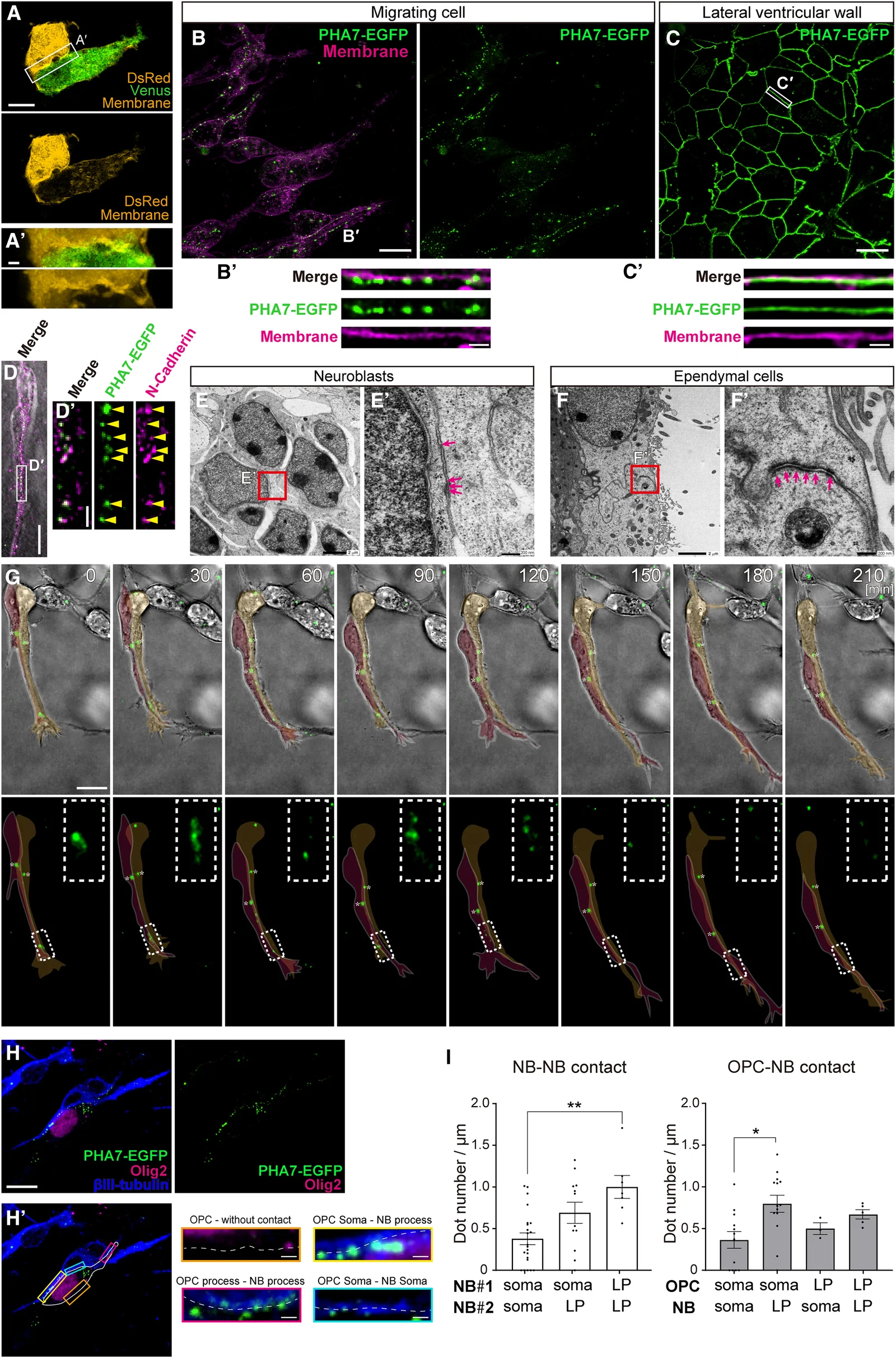
OPCs form spatiotemporally dynamic adherens junctions with neuroblasts (A) Super-resolution image of contact between an oligodendrocyte progenitor cell (OPC) (green) and a neuroblast (yellow) in *Sox10-Venus;Dcx-DsRed* V-SVZ explant cultures embedded in Matrigel. Plasma membranes were labeled with PlasMem Bright Red (yellow). (A′) shows higher magnification of the boxed region. (B and C) V-SVZ explants from *R26R-PHA7-EGFP;Nestin-Cre* mice. PHA7-EGFP (green) and plasma membranes (magenta) in cells migrating from the explant (B) and at the ventricular surface (C). (B′) and (C′) show higher magnification of the boxed regions. (D) Migrating cells in *R26R-PHA7-EGFP;Nestin-Cre* explant cultures immunostained for N-cadherin (magenta). (D′) shows higher magnification of the boxed region. Yellow arrowheads indicate PHA7-EGFP puncta (green) colocalized with N-cadherin. (E and F) Transmission electron microscopy images of chain-forming neuroblasts (E) and ependymal cells (F) in wild-type mouse V-SVZ. (E′) and (F′) show higher magnification of the boxed regions. Arrows indicate adherens junctions with electron-dense plaques. (G) Time-lapse imaging of PHA7-EGFP puncta in migrating cells. Cell pairs are highlighted with semi-transparent yellow and red overlays. Insets (bottom parts of the images) show higher magnification of the dotted regions. Asterisks indicate intracellular puncta (centrosomes within swellings). (H) Immunostaining for Olig2 (magenta) and βIII-tubulin (blue) in explant cultures. Subregions of the OPC membranes exhibiting distinct contact patterns with neuroblasts (outlined in H′) are magnified in the right images. Dotted lines indicate boundaries between contacting cells as defined by PlasMem Bright Red staining. (I) Quantification of PHA7-EGFP puncta density at neuroblast-neuroblast (NB-NB, left) and neuroblast-OPC (NB-OPC, right) contact sites. Contact types were classified based on the contacting subcellular domain, defined as either the soma or the leading process (LP), as indicated. Each dot represents one contact region. 34 OPC-NB contact sites (12 OPC soma-NB soma, 14 OPC soma-NB process, 3 OPC process-NB soma, and 5 OPC process-NB process) and 41 NB-NB contact sites (22 NB soma-NB soma, 12 NB soma-NB process, and 7 NB process-NB process) derived from 14 OPCs and 76 NBs from 2 independent experiments were analyzed. Data are mean ± SEM. ∗*p* < 0.05, ∗∗*p* < 0.01; one-way ANOVA followed by Tukey’s multiple comparisons test. Scale bars: 10 μm: (A–D), (G), and (H); 2 μm: (E) and (F); 1 μm: (A′–D′) and magnified subregions in (H); 200 nm: (E′) and (F′).

To characterize the physical nature of OPC-neuroblast interactions, we performed high-resolution imaging of fluorescently labeled plasma membranes at contact sites. The membranes of Venus^+^ OPCs were contiguous with those of DsRed^+^ neuroblasts (Figure 5A and Video S3), reminiscent of the homologous interactions observed among neuroblasts.^38^ Since both neuroblasts and OPCs express N-cadherin,^22^^,^^39^ a key component of AJs, we hypothesized that AJ-like structures might mediate these dynamic intercellular contacts.

Video S3. Three-dimensional reconstruction of contact between an OPC and a neuroblast, related to Figure 5A Three-dimensional reconstructed super-resolution image of a contact between an oligodendrocyte progenitor cell (OPC; green) and a neuroblast (yellow) in *Sox10-Venus;Dcx-DsRed* V-SVZ explant cultures embedded in Matrigel. Plasma membranes were labeled with PlasMem Bright Red (yellow). The relative positions of the cells are shown in the first half, and a magnified view of the boxed contact region is shown in the second half. The scale is indicated within the boxed inset (in μm).

To visualize AJs during chain migration, we utilized *R26R-PHA7-EGFP;Nestin-Cre* mice, in which Nestin-derived lineages—including both neuroblasts and OPCs—express a GFP-tagged version of Plekha7 (PHA7),^40^^,^^41^ a key component of the AJ complex.^42^ In super-resolution imaging of the V-SVZ tissue embedded in Matrigel (Figures 5B–5D), ependymal cells, which harbor strong AJs between them as typically observed among epithelial cells,^43^ exhibited contiguous linear PHA7-EGFP signals at the ventricular surface (Figure 5C). In contrast, punctate PHA7-EGFP signals were observed at the interfaces between cells within migratory chains (Figure 5B). These observations well reflected *in vivo* observations of the V-SVZ by transmission electron microscopy: neuroblasts in chains possess small, discontinuous AJs that are less electron-dense than typical AJs between ependymal cells^44^ (Figures 5E and 5F). We also noted a single intracellular punctum in each cell, likely representing centriole-associated signals as previously described.^42^ The punctate PHA7-EGFP signals on the plasma membranes, but not the intracellular signals, were largely co-localized with endogenous N-cadherin (Figure 5D), and rapidly disappeared upon treatment with EGTA, a calcium chelator (Figure S4A), suggesting that these puncta represent calcium-dependent, cadherin-based adhesive interactions.

Live-cell imaging further highlighted the dynamic nature of these junctions. Linear PHA7-EGFP signals in the ependyma remained stable over 16 h (Figure S4B and Video S4), whereas punctate signals at the membrane within migratory chains were highly dynamic: preexisting PHA7 puncta disappeared as one of the contacting cells moved forward, and new puncta formed following somal translocation (Figure 5G and Video S5). Similar punctate AJs were observed between Olig2^+^ OPCs and βIII-tubulin^+^ neuroblasts (Figure 5H). In addition, temporally dynamic AJs were detected in an OPC in *Olig2-CreER;R26R-PHA7-EGFP* mice within a migratory chain (Figure S4C), supporting the model that OPCs form heterotypic AJs with neuroblasts to facilitate their migration. Notably, in both OPC-neuroblast and neuroblast-neuroblast interactions, the frequency of these AJ-like structures was significantly higher at interfaces involving leading processes compared to soma-soma contact sites (Figures 5I and S4D).

Video S4. Stable PHA7-EGFP signals in the ventricular wall, related to Figure S4B Time-lapse imaging of V-SVZ explant cultures from *R26R-PHA7-EGFP;Nestin-Cre* mice acquired at 30-min intervals. PHA7-EGFP (green) signals at the ventricular surface are shown.

Video S5. Dynamic PHA7-EGFP signals in migrating cells, related to Figure 5G Time-lapse imaging of V-SVZ explant cultures from *R26R-PHA7-EGFP;Nestin-Cre* mice acquired at 30-min intervals. Migrating cells are shown, with the left panel displaying paired cells highlighted by semi-transparent yellow and red overlays and the right panel showing the same field without highlighting. Asterisks indicate intracellular puncta (centrosomes within swellings). Scale bars: 10 μm.

Together, these results suggest that V-SVZ-derived OPCs facilitate their migration toward cortical lesions by utilizing highly motile neuroblasts through spatially discontinuous and temporally dynamic contacts, and subsequently differentiate into oligodendrocytes that contribute to remyelination.

## Discussion

Postnatal OPC recruitment to sites of brain injury has been well documented; however, the mechanisms enabling efficient long-distance migration from the V-SVZ remain poorly defined. Here, using neonatal cortical injury models and three-dimensional culture systems, we demonstrated that postnatal V-SVZ-derived OPCs migrated more efficiently through direct physical interactions with migrating neuroblasts. These heterotypic contacts form dynamic, discontinuous AJs and are associated with OPC motility. Our findings identify actively migrating neuroblasts as an unexpected cellular scaffold that facilitates long-range OPC recruitment in the postnatal brain.

Unlike embryonic OPCs, which preferentially migrate along blood vessels,^28^ postnatal V-SVZ-derived OPCs did not appear to primarily use the vasculature as a migratory scaffold under the experimental conditions used in this study (Figure 2). This difference may reflect progressive vascular ensheathment by astrocytic endfeet during development, limiting access to endothelium.^45^ In injured tissue, swollen endfeet of reactive astrocytes^46^ may further isolate the vasculature. Instead, V-SVZ-OPCs frequently aligned with radial glial fibers (Figure 2)—consistent with earlier predictions.^47^ Because radial glial fibers persist longer after neonatal injury than in the adult brain, this mechanism may be particularly relevant in the neonatal context. Importantly, OPCs directly contacted migrating neuroblasts during their migration toward cortical lesions, suggesting a functional interaction between the two cell populations. Such heterotypic interactions were not observed in the postnatal intact brain, where the majority of neuroblasts and OPCs migrate in different directions toward distinct destinations.^5^^,^^7^ In contrast, these contacts were frequently observed in three-dimensional cultures, in which both cell types emerge from the explant and migrate radially away from it along similar trajectories. Although neuroblast-mediated motility occurred independently of radial glial scaffolds *in vitro* (Figure 4), convergence toward a common lesion site *in vivo*, together with shared use of radial glial structures, may increase the likelihood of OPC-neuroblast encounters within otherwise sparsely distributed regions.

Notably, while V-SVZ-OPC migration was enhanced upon contact with neuroblasts, neuroblast motility remained largely unaffected by heterotypic interactions (Figure 3C). In contrast, homologous interactions among neuroblasts leading to chain formation significantly increased migration speed (Figure 4F), underscoring the intrinsic efficiency of collective neuroblast migration. Neuroblasts are known to remodel their surrounding microenvironment to navigate complex interstitial spaces,^48^^,^^49^^,^^50^ a process further amplified by chain organization.^51^ Interestingly, embryonic ventral germinal zone-derived earlier OPCs exhibit Semaphorin6a/b-Plexin A3-mediated contact repulsion with interneurons during cortical migration,^52^ whereas this repulsive interaction is reduced in later embryonic stages. This developmental shift suggests that interactions between OPCs and neuroblasts are context-dependent and may be modulated according to the structural complexity of the surrounding tissue. We did not observe OPC-OPC contacts, which may reflect the relatively sparse distribution of OPCs in the V-SVZ.^53^ Although it remains unclear whether OPCs independently possess comparable environment-modifying capabilities, our findings suggest that heterotypic contact with migrating neuroblasts provides a functional advantage for OPC migration in injured tissues and represents a form of heterotypic collective migration.

Using PHA7-EGFP mice, we visualized the dynamics of cadherin-associated AJs between OPCs and neuroblasts, as well as among neuroblasts themselves (Figure 5). These junctions were punctate and spatially discontinuous, consistent with ultrastructural observations,^38^ and were highly dynamic, forming and dissolving in coordination with cell movement. The transient and spatially discontinuous organization of these AJs contrasts with classical collective migration, in which junctions are typically stable and continuous.^41^^,^^53^ Because excessively stable adhesion can impede cell motility,^39^^,^^51^^,^^54^ the dynamic and punctate nature of these contacts may represent a structural adaptation that balances cohesion with migratory flexibility.

How does neuroblast contact accelerate OPC migration? N-cadherin-mediated adhesion may promote cytoskeletal remodeling^55^ or activate RhoA signaling pathways,^22^ mechanisms that could similarly enhance OPC motility upon heterotypic contact. In addition to biochemical signaling, these contacts may provide mechanical cues. Recent studies have shown that mechanosensitive pathways involving TMEM63b^56^ and PIEZO1^57^ contribute to neuronal migration. The preferential localization of AJs at dynamically extending and retracting leading processes (Figure 5I) supports the possibility that these contacts act as localized mechanical cues that may activate mechanosensitive signaling pathways. Because both neuroblasts and OPCs express multiple adhesion molecules beyond N-cadherin,^22^^,^^39^ additional molecular interactions may further modulate this cooperative migration. Although further studies are required to delineate the signaling mechanisms governing these dynamic AJs and their possible contribution to enhanced migration, our findings shed light on a new aspect of the regulation of intercellular coordination in progenitor migration.

Demyelination and traumatic brain injury trigger the proliferation and mobilization of OPCs within the V-SVZ.^5^^,^^8^^,^^58^^,^^59^ While pOPCs serve as the principal source for local remyelination, V-SVZ-OPCs may provide a supplementary pool, particularly in extensive injuries where local pOPC populations have been depleted^60^ or in deep-seated lesions. Such severe injuries frequently involve concurrent neuronal loss, creating a context in which coordinated recruitment of OPCs and neuroblasts to the same lesion site may be advantageous. Although further investigation is needed, the heterotypic migratory coupling described here suggests a potential strategy for enhancing OPC recruitment to promote myelin repair.

### Limitations of the study

In this study, we found that contact with neuroblasts is associated with enhanced OPC migration. However, the respective contributions of radial glial fibers and blood vessels to OPC migration were not directly assessed. In addition, the mechanisms by which AJs directly contribute to enhanced migration remain to be clarified. Addressing these questions in future studies will provide a deeper understanding of the mechanisms underlying OPC migration.

## Resource availability

### Lead contact

Further information and requests for resources and reagents should be directed to and will be fulfilled by the lead contact, Naoko Kaneko (nkaneko@mail.doshisha.ac.jp).

### Materials availability

Materials generated in this study are available from the lead contact upon request, subject to a completed materials transfer agreement.

### Data and code availability

• All data reported in this paper will be shared by the lead contact upon request.
• This paper does not report original code.
• Any additional information required to reanalyze the data reported in this paper is available from the lead contact upon request.

## Acknowledgments

We thank Dr. Rüdiger Klein (Max Planck Institute) for providing *Nestin-Cre* mice; Dr. Masahiro Yamaguchi (Kochi Medical School, Japan) for providing *R26R-tdTomato* mice; Dr. Qiang Lu (10.13039/100008307Beckman Research Institute of the City of Hope) for providing *Dcx-DsRed* mice; Mutant Mouse Resource and Research Center (MMRRC) for providing *Dcx-EGFP* mice, and 10.13039/501100006264RIKEN BDR Laboratory for Animal Resources and Genetic Engineering for providing R26R-*PHA7-EGFP* mice. This work was supported by research grants from 10.13039/501100001691Japan Society for the Promotion of Science (JSPS) 10.13039/501100001691KAKENHI (20H03565, 21H05106, 23H02579, and 23K27270 to N.K.; 24K18282 to Y.S.; and 25H01040 and 25H02507 to K.S.), Core-to-Core Program (JPJSCCA20230007 to K.S.), JST FOREST Program (JPMJFR2146 to N.K.), the 10.13039/100009619Japan Agency for Medical Research and Development (AMED) (24gm1210007 to K.S.), Grant-in-Aid for Outstanding Research Group Support Program in 10.13039/501100008883Nagoya City University (2401101 to K.S.) and the Valencian Council for Education, Culture, University and Employment (CIPROM/2023/053) to V.H.-P.

## Author contributions

Y.S., K.S., and N.K. designed the study. Y.S., N.I., E.W.K.U., M.H., K.K., and N.K. performed the experiments. Y.S., E.W.K.U., M.H., V.H.-P., and N.K. analyzed the data. C.A., S.S., H.T., and H.O. provided critical reagents and contributed to data interpretation and manuscript discussion. Y.S., K.S., and N.K. wrote the manuscript. All authors read and approved the final manuscript.

## Declaration of interests

The authors declare no competing interests.

## Declaration of generative AI and AI-assisted technologies in the writing process

During the preparation of this work the authors used ChatGPT (OpenAI) and Gemini (Google) in order to improve the grammar and clarity of the manuscript. After using these tools, the authors reviewed and edited the content as needed and take full responsibility for the content of the publication.

## STAR★Methods

### Key resources table

**Table:** 

REAGENT or RESOURCE	SOURCE	IDENTIFIER
**Antibodies**		

Rabbit anti-Olig2	IBL	#18953; RRID: AB_3697072
Mouse anti-S100β	Sigma-Aldrich	#2532; RRID: AB_477499
Rabbit anti-NeuN	Abcam	#EPR12763; RRID: AB_2532109
Mouse anti-APC	Millipore	#OP80; RRID: AB_2057371
Rabbit anti-MBP	Abcam	#ab40390; RRID: AB_1141521
Armenian Hamster anti-CD31	Developmental Studies Hybridoma Bank	#2H8; RRID: AB_2161039
Chicken anti-Nestin	Aves Labs	#NES; RRID: AB_2314882
Guinea pig anti-Dcx	Millipore	#AB2253; RRID: AB_1586992
Chicken anti-Neurofilament	Novus Biologicals	#NB300-222; RRID: AB_10002431
Mouse anti-βIII-tubulin	Sigma-Aldrich	#T8660; RRID: AB_477590
Mouse anti-N-Cadherin	BD Biosciences	#610920; RRID: AB_398236
Donkey Anti-Mouse IgG H&L (Alexa Fluor® 405)	Abcam	#ab175658; RRID: AB_2687445
Alexa Fluor 488 donkey anti-mouse IgG (H+L)	Thermo Fisher Scientific	#A21202; RRID: AB_141607
Alexa Fluor 647 donkey anti-mouse IgG (H+L)	Thermo Fisher Scientific	#A31571; RRID: AB_162542
Donkey Anti-Rabbit IgG H&L (Alexa Fluor® 647)	Abcam	#ab175649; RRID: AB_2715515
Alexa Fluor 647 donkey anti-rabbit IgG (H+L)	Thermo Fisher Scientific	#A31573; RRID: AB_2536183
Cy3-AffiniPure donkey anti-chicken IgY (IgG) (H+L)	Jackson ImmunoResearch	#703-165-155; RRID: AB_2340363
Goat Anti-Guinea pig IgG H&L (Alexa Fluor® 405)	Abcam	#abab175678; RRID: AB_2827755
Alexa Fluor 647-AffiniPure Goat Anti-Armenian Hamster IgG (H+L)	Jackson ImmunoResearch	#127-605-160; RRID: AB_2339001

**Chemicals, peptides, and recombinant proteins**		

Hoechst 33342	Thermo Fisher Scientific	#H1399
PlasMem Bright Red	Dojindo	#P505
Paraformaldehyde (PFA)	Wako	#162-16065
Fast green	Sigma-Aldrich	#F7252
Tamoxifen	Sigma-Aldrich	#T5648
Sesame oil	Nacalai Tesque	#25620-65
DMSO	Wako	#049-07213
Ethanol	Nacalai Tesque	#08948-25
HistoVT One	Nacalai Tesque	#06380-76
Normal donkey serum	Sigma-Aldrich	#S30-100ML
Neurobasal medium	Thermo Fisher Scientific	#21103049
Trypsin-EDTA	Wako	#201-16945
Leibovitz’s L-15	Wako	#044-29765
DNase I	Sigma-Aldrich	#10104159001
Matrigel	Corning	#354234
MACS® NeuroBrew®-21	Miltenyi Biotec	#130-093-566
D-MEM (Low Glucose) with L-Glutamine and Phenol Red	Wako	#041-29775
Fetal Bovine Serum (FBS)	Corning	#35-010-CV
GlutaMAX Supplement	Thermo Fisher Scientific	#35050061
Penicillin-Streptomycin	Wako	#168-23191

**Critical commercial assays**		

PureLink HiPure Plasmid Maxiprep Kit	Thermo Fisher Scientific	#K210007

**Experimental models: Organisms/strains**		

Mouse: ICR	Japan SLC	N/A
Mouse: C57BL6/J	Japan SLC	N/A
Mouse: Rosa26R-tdTomato	The Jackson Laboratory	#007914; RRID: IMSR_JAX:007914
Mouse: Dcx-EGFP	Gong et al.^35^ Mutant Mouse Resource and Research Center (MMRRC)	#000244-MU; RRID: MMRRC_000244-MU
Mouse: Dcx-DsRed	Wang et al.^37^ gift from Dr. Qiang Lu	N/A
Mouse: Sox10-Venus	Shibata et al.^36^	N/A
Mouse: Nestin-Cre	Tronche et al.^40^ gift from Dr. Rüdiger Klein	RRID: IMSR_JAX:003771
Mouse: R26R-PHA7-EGFP	Shioi et al.^41^ RIKEN BDR Laboratory for Animal Resources and Genetic Engineering (http://www.clst.riken.jp/arg/reporter_mice.html)	#CDB0226K
Mouse: Olig2-CreER^TM^	Takebayashi et al.^23^	N/A

**Recombinant DNA**		

pcDNA3.1-mCherry	Addgene, Kleaveland et al.^61^	#128744; RRID: Addgene_128744
pCAGGS-EmGFP-miR-LacZ	Ota et al.^62^	N/A
pCAGGS-tdTomato-miR-LacZ	Jinnou et al.^22^	N/A
pPyCAG-Cre-IRESpuropA	Tashiro et al.^63^ RIKEN BRC	#RDB09596
pCAGGS-Cre	This paper	N/A

**Software and algorithms**		

ImageJ/Fiji	Schindelin et al.^64^	https://imagej.net/software/fiji/
GraphPad Prism 10	GraphPad Software	https://www.graphpad.com/

**Other**		

Electroporator	NepaGene	NEPA21
LSM880	Carl Zeiss	N/A
LSM710	Carl Zeiss	N/A
FV3000	Evident	N/A
FV4000	Evident	N/A
Nikon Ax	Nikon	N/A
JEM-1400Plus	JEOL	N/A

### Experimental model and study participant details

All animal experiments were performed in accordance with the guidelines and regulations of Doshisha University (approval number: A26055).

*Wild-type* (WT) ICR mice were purchased from Japan SLC (Shizuoka, Japan). *Sox10-Venus*^36^ and *Olig2-CreER*^*TM*^ mice^23^ were described previously. *Dcx-DsRed* transgenic mice^37^ were provided by Dr. Qiang Lu (Beckman Research Institute of the City of Hope). *Rosa26R (R26R)-tdTomato* mice were provided by Dr. Masahiro Yamaguchi (Kochi Medical School, Japan). *Nestin-Cre mice*^40^ were provided by Dr. Rüdiger Klein (Max Planck Institute). *R26R-PHA7-EGFP* mice^41^ (CDB0226K) were provided by RIKEN BDR Laboratory for Animal Resources and Genetic Engineering (http://www.clst.riken.jp/arg/reporter_mice.html). *Dcx-EGFP* mice^35^ (#000244-MU) were provided by the Mutant Mouse Research Resource Center (MMRRC). *Sox10-Venus* mice were crossbred with *Dcx-DsRed* mice and *Olig2-CreER*^*TM*^ mice were crossed with *R26R-tdTomato* and then their offspring were crossed with *Dcx-EGFP* lines to obtain *Olig2-CreER;R26R-tdTomato;Dcx-EGFP* triple-transgenic mice. For adherens junction imaging, *R26R-PHA7-EGFP* mice were intercrossed with either *Nestin-Cre* or *Olig2-CreER*^*TM*^ lines. The genotypes were confirmed by PCR analysis of tail or ear biopsies.

### Method details

#### Cryogenic injury of the neonatal cortex

For *Olig2-CreER;R26R-tdTomato* or *Olig2-CreER;R26R-tdTomato;Dcx-EGFP* mice, tamoxifen dissolved in vehicle (90% sesame oil, 6% ethanol, and 4% DMSO) was administered by intraperitoneal (i.p.) injection (200 μg/mouse) 4 hours to 2 days prior to injury. At postnatal days (P) 2–4, mice were anesthetized via hypothermia, and the parietal skull was exposed through a scalp incision. A focal cryogenic injury was induced by applying a pre-cooled hex wrench (1.5-mm diameter), chilled in liquid nitrogen, to the surface of the right skull (2.0 mm anterior and 1.2 mm lateral to the lambda). The wrench was placed there for three 10-second intervals. Following the procedure, the scalp was immediately sutured, and the mice were returned to their home cages.

#### Plasmid construction

The pCAGGS-Cre vector was constructed using the Gateway cloning system. The Cre fragment was amplified from pPyCAG-Cre-IRESpuropA^63^ using KOD-Plus-Neo DNA polymerase (TOYOBO, #KOD-401) with the following primers: forward, 5′-CACCATGTCCAATTTACTGACCGTACACC-3′; reverse, 5′-CTAATCGCCATCTTCCAGCAGGCGC-3′. The amplified fragment was cloned into pENTR/D-TOPO (Invitrogen). Then the DNA cassette was cloned into a modified pCAGGS vector using Gateway LR Clonase II Enzyme Mix (Thermo Fisher Scientific, #11791020).

#### V-SVZ electroporation

P0 mice were anesthetized via hypothermia and secured in a stereotaxic injection apparatus (David Kopf Instruments). The pCAGGS-EmGFP-miR-LacZ^62^ or pcDNA3.1-mCherry^61^ expression vectors were injected into the right lateral ventricle (1.8 mm anterior and 1.2 mm lateral to the lambda, 1.8 mm depth). Following injection, electroporation was performed by delivering three electrical pulses (70 V, 30.0 ms) using a Super Electroplater NEPA21 Type II (Nepa Gene) equipped with 7-mm tweezer electrodes (CUY650P7, Nepa Gene).

#### Slice live imaging

Injured *Olig2-CreER;R26R-tdTomato* or *Olig2-CreER;R26R-tdTomato;Dcx-EGFP* mice were deeply anesthetized with isoflurane and the brains were rapidly removed. The brains were secured to the specimen holder and stabilized by embedding in 3% NuSieve GTG agarose (Lonza). Coronal slices (200 μm thick) were prepared using a vibratome (VT1200S, Leica) in ice-cold DMEM (Wako). The slices were then maintained under constant perfusion with 35–37°C artificial cerebrospinal fluid (124 mM NaCl, 24 mM NaHCO_3_, 12.5 mM glucose, 5 mM HEPES, 2.5 mM KCl, 1.2 mM NaH_2_PO_4_, 2 mM CaCl_2_, 2 mM MgSO_4_ [pH 7.4]) bubbled with 95% O_2_ / 5% CO_2_. Time-lapse images of the injured or intact cortex were captured every 12 minutes for 80 time points using a Zeiss confocal microscope (LSM710, Carl Zeiss) equipped with a 20× objective lens (NA 0.8).

#### V-SVZ explant culture

V-SVZ tissues from neonatal mice (P0–P6) were isolated and dissected into pieces (100–200 μm in diameter) in cold Leibovitz’s L-15 Medium (Wako). For *Olig2-CreER;R26R-PHA7-EGFP* cultures, tamoxifen was injected i.p. at P2, and the V-SVZ was isolated at P7. Explants were embedded in 70% Matrigel (Corning) diluted with L-15 medium on glass-bottom dishes (Matsunami, D141400 or D11140H) and allowed to polymerize at 37°C, 5% CO_2_. For dissociated cell cultures, the isolated V-SVZ explants were incubated in 0.25% trypsin-EDTA for 10 minutes at RT and then mechanically dissociated with D-MEM containing 5% FBS (Corning) / DNase I (Sigma) and passed through a 70 μm cell strainer (Corning, #352350). Cells were collected by centrifugation at 1,000 xg for 10 minutes at 4°C. The resulting cell pellet was resuspended with culture medium and plated onto glass coverslips pre-coated with Matrigel (diluted 1:80 in L-15 medium).

Cells were cultured in Neurobasal medium (Thermo Fisher Scientific) supplemented with 10% FBS, 2% MACS NeuroBrew-21 (Miltenyi Biotec), 2 mM GlutaMAX Supplement (Thermo Fisher Scientific), and 1% penicillin-streptomycin (Wako). To label plasma membranes, cells were incubated with PlasMem Bright Red (Dojindo) (1:100) for 15 minutes prior to fixation or imaging.

#### Live cell imaging of V-SVZ explant culture

Live imaging was performed in a stage-top incubator maintained at 37°C and 5% CO_2_. For *Sox10-Venus;Dcx-DsRed* V-SVZ cultures, a confocal microscope (FV3000, Evident or LSM880, Carl Zeiss) was used, with images acquired every 4 or 10 minutes with a 20× objective lens (NA 0.8). For PHA7-EGFP imaging, a Nikon AX confocal microscope with a 60× oil-immersion objective lens (NA 1.42) was used, with images acquired every 30 minutes. Super-resolution images were acquired using the Nikon Spatial Array Confocal (NSPARC) detector.

#### Immunohistochemistry / immunocytochemistry

For tissue preparation, mice were anesthetized via hypothermia/isoflurane inhalation or an intraperitoneal injection of anesthetic mixture (0.75 mg/kg medetomidine, 4.0 mg/kg midazolam, and 5.0 mg/kg butorphanol). Mice were transcardially perfused with phosphate-buffered saline (PBS), followed by 4% paraformaldehyde (PFA) in 0.1 M phosphate buffer (PB). The brains were carefully removed and post-fixed in the same fixative at 4°C overnight. Coronal sections were prepared using a vibratome at a thickness of 70 μm for neonates and 50 μm for P30 mice.

For immunostaining, the sections were incubated in blocking buffer (10% normal donkey serum and 0.4% Triton X-100 in PBS) at room temperature (RT) for 1 hour, followed by incubation with primary antibodies diluted in blocking buffer at 4°C overnight. The sections were then washed three times for 10 minutes each with PBS-T (0.2% Triton X-100) and incubated with fluorescently labeled secondary antibodies (1:500) with or without Hoechst 33342 in blocking buffer at RT for 3 hours.

For immunocytochemistry, cultured cells were rinsed with PBS and fixed with 4% PFA. For cultured cells in Matrigel, heat-induced epitope retrieval was performed using HistoVT One (pH 7.0; Nacalai Tesque) at 85°C for 20 minutes prior to immunostaining. Subsequent staining protocols followed the immunohistochemistry procedure described above. Images were acquired using either the confocal microscopes described above or a FV3000 (Evident) using 20× objective lens (NA 0.8) or 60× oil-immersion objective lens (NA 1.42).

For primary antibodies: anti-Olig2 (1:500, IBL), anti-S100β (1:500, Sigma-Aldrich), anti-NeuN (1:1000, Abcam), anti-APC (CC-1) (1:500, Calbiochem), anti-MBP (1:100, Abcam), anti-CD31 (1:100, Developmental Studies Hybridoma Bank), anti-Nestin (1:1000, Aves Labs), anti-Dcx (1:1000 for tissue, 1:2000 *in vitro*, Millipore), anti-Neurofilament (1:1000, Millipore), anti-βIII-tubulin (1:500, Sigma-Aldrich), and anti-N-cadherin (1:2000, BD Biosciences) were used.

#### Image analysis

All images were processed as maximum intensity projections using Fiji/ImageJ.^64^ For PHA7-EGFP analysis, puncta number and intensity were manually measured at continuous contact sites longer than 3 μm. Background subtraction and Gaussian filtering were applied uniformly across all datasets. For calculation of cell migration speed, cells were tracked using Manual Tracking plugin in Fiji/ImageJ. The directionality index was calculated by dividing net displacement by total migration path length. For brain-slice live imaging experiments, only tdTomato-positive cells exhibiting a bipolar morphology and directional migration were included in the analysis. On the ipsilateral side, cells migrating toward the lesion were analyzed, whereas on the contralateral side, cells migrating toward the corresponding dorsal cortex were analyzed. To evaluate the effect of neuroblast contact on OPC, migration speeds were calculated specifically for individual OPCs that exhibited both “contact” and “without-contact” phases during the recording period.

#### Transmission electron microscopy

Adult *wild-type* mice were fixed by transcardiac perfusion with 2% PFA and 2.5% glutaraldehyde containing 0.1 M PB. The brains were extracted and post-fixed for 48 h in the same fixative at 4°C and sliced into 200 μm coronal sections using a vibratome. The sections were treated with 4% OsO_4_ in 0.1 M PB for 90 minutes, dehydrated in graded concentrations of ethanol and propylene oxide and embedded in epoxy resin (Durcupan, Sigma Aldrich) for 72 h in a 60°C oven to achieve complete polymerization of the resin. Serial semi-thin sections (1.5-μm-thick) were cut with an ultramicrotome (UC6, Leica) using a diamond knife (Histo, Diatome, Hatfield, PA, USA), and stained lightly with 1% toluidine blue. After acquisition of the images with a transmitted light microscope (Olympus BX51, Evident, Tokyo, Japan), the semi-thin sections were glued to Durcupan blocks and detached from the glass slide by repeated freezing in liquid nitrogen and thawing. Ultra-thin sections (60–70-nm-thick) were prepared from the semi-thin sections using an ultramicrotome (UC6, Leica) and a diamond knife (SYM2045, Syntek, Kanagawa, Japan), and stained with 2% uranyl acetate for 15 minutes and with modified Sato's lead solution (Reynolds' solution) for 5 minutes. Images were captured using a transmission electron microscope (JEM-1400Plus, JEOL, Tokyo, Japan) equipped with a digital camera.

### Quantification and statistical analysis

Statistical analysis was conducted using GraphPad Prism, with significance set at *p* < 0.05. Error bars in bar graphs represent the standard error of the mean (SEM) whereas box plots show the median, upper/lower quartiles (box), and min/max values (whiskers). For comparisons between two independent groups, Welch’s unpaired t-test was used. For comparisons among three or four independent groups, One-way ANOVA followed by Tukey’s multiple comparisons test was used. Significance levels in figures are denoted by (ns), (∗), (∗∗) and (∗∗∗) for not significant, *p* < 0.05, *p* < 0.01 and *p* < 0.001, respectively.
