# Supplementary material for: Dynamic interactions with neuroblasts promote oligodendrocyte progenitor migration to injured cortex
Source: iScience. 2026 Aug 24;29(9):117318. doi: 10.1016/j.isci.2026.117318 (PMC13542502; doi:10.1016/j.isci.2026.117318)
Supplement: Supplementary file 1 [file mmc1.pdf]

iScience, Volume *29*

Supplemental information

Dynamic interactions with neuroblasts promote oligodendrocyte progenitor migration to injured cortex

Yuriko Sobu, Nodoka Ito, Edward William Ko Uy, Mayaka Hashimoto, Kazuya Kuboyama, Chihiro Akazawa, Shinsuke Shibata, Hirohide Takebayashi, Hideyuki Okano, Vicente Herranz-Pérez, Kazunobu Sawamoto, and Naoko Kaneko

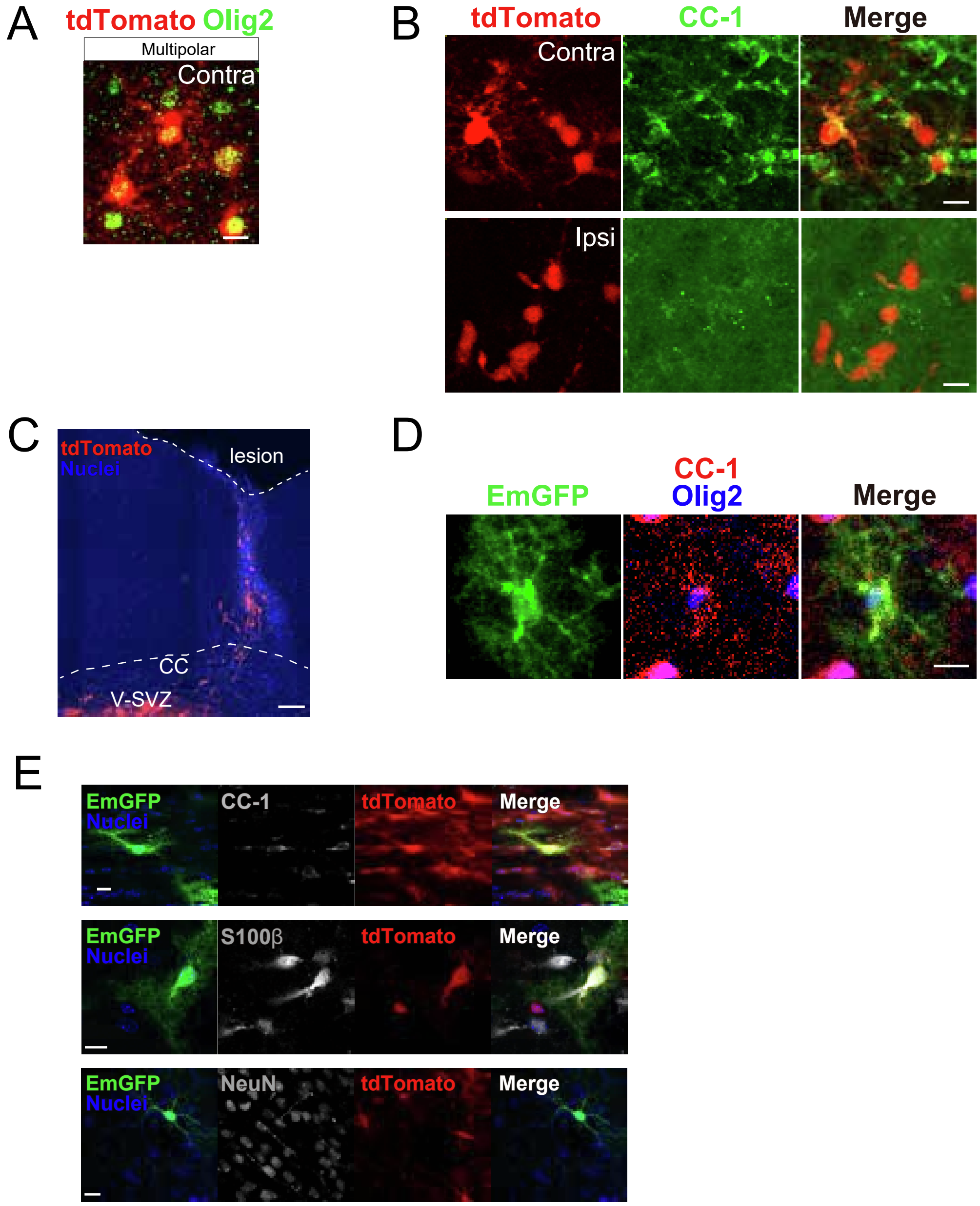

Figure S1. Properties of tdTomato+ cells in the intact and injured neonatal brain, related to Figure 1.

(A, B) Coronal brain sections from postnatal day (P) 6 Olig2-CreER;R26R-tdTomato mice subjected to cortical lesion at P4. The sections were immunostained for Olig2 (green) or APC (green). tdTomato+Olig2+ oligodendrocyte progenitor cells (OPCs) with multipolar morphology in contralateral neocortices are shown (A). OPCs in contralateral neocortex that were positive for CC-1 (B, top) and OPCs around the lesion that were negative for CC-1 (B, bottom) are shown.

(C) tdTomato-labeled V-SVZ-derived cells 4 weeks after injury. The V-SVZ, the border of the corpus callosum (CC), and the lesion site are indicated in the image.

(D) Coronal brain section from a P30 wild-type mouse electroporated with a pCAGGS-EmGFP vector at P0 and subjected to cortical lesion at P3, immunostained for CC-1 (red) and Olig2 (blue).

(E) Fate mapping of EmGFP+ (green) cells labeled by electroporation in the ipsilateral neocortices of Olig2-CreER;R26R-tdTomato mice. EmGFP+ (green);tdTomato+ (red) cells in the ipsilateral neocortex at P30 were immunostained for APC, S100β, or NeuN (gray).

Scale bars: 10 µm: A, B, D; 20 µm: E; 100 µm: C.

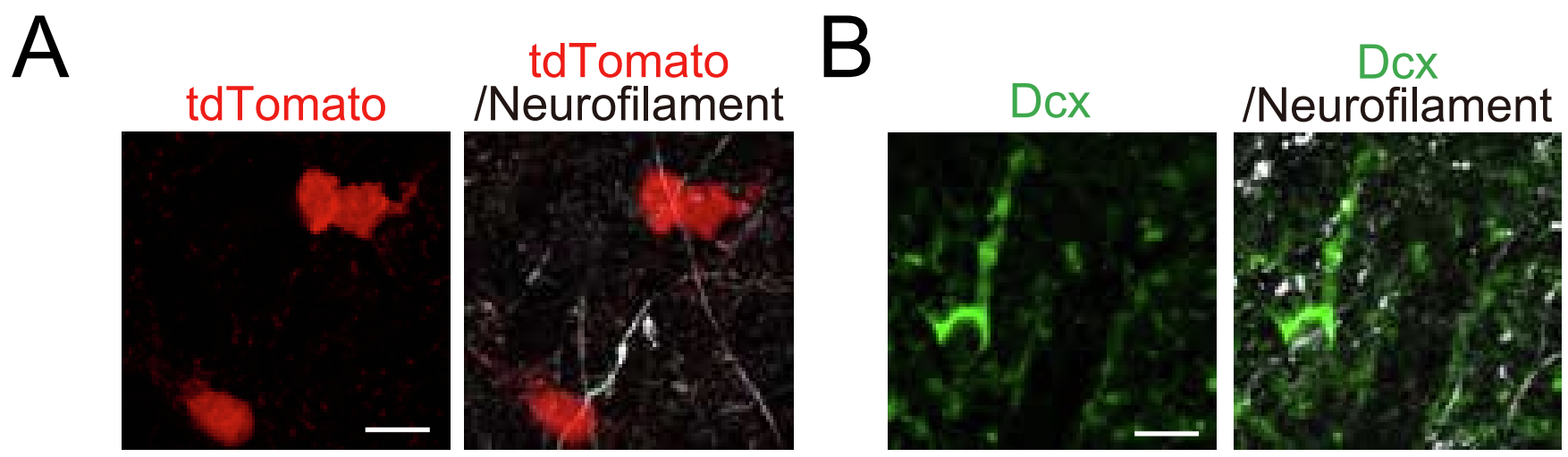

Figure S2. Bipolar-like OPCs and neuroblasts are not aligned with axons, related to Figure 2.

(A, B) Coronal brain sections from postnatal day (P) 6 Olig2-CreER;R26R-tdTomato mice subjected to cortical lesion at P4, immunostained for the neuroblast marker Dcx (green) and the axonal marker Neurofilament (gray). Bipolar-like tdTomato+ oligodendrocyte progenitor cells (OPCs, red) (A) and Dcx+ neuroblasts (B) are shown in the injured neocortex. Scale bars: 10 μm: A, B.

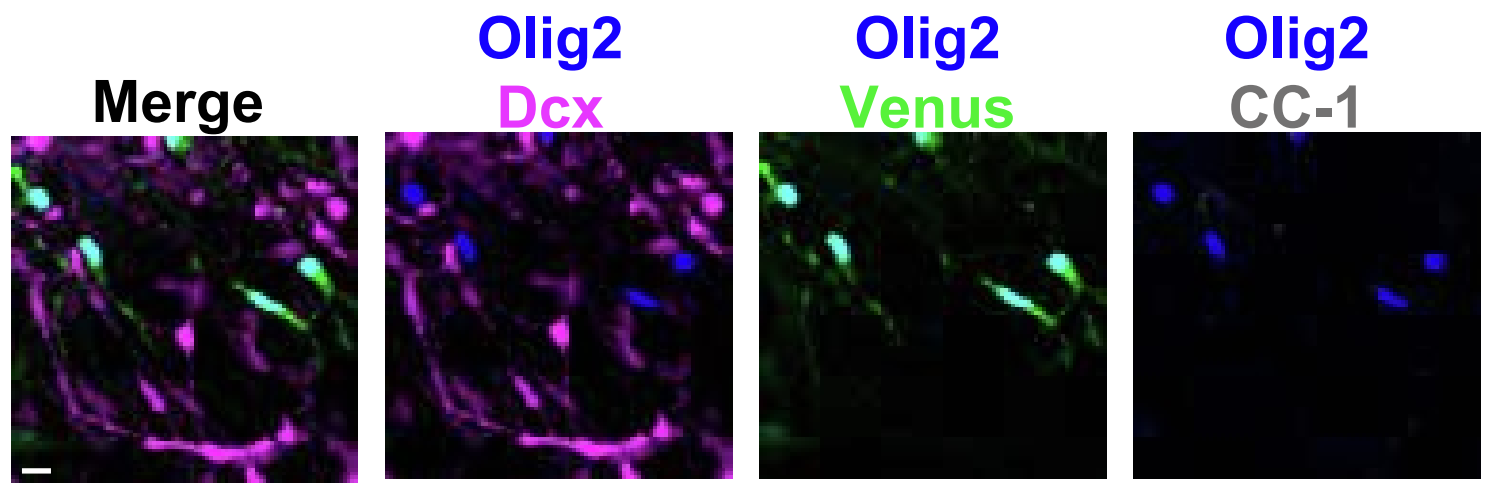

Figure S3. OPCs from Sox10-Venus V-SVZ culture remain immature, related to Figure 4.

Dissociated V-SVZ culture from Sox10-Venus;Dcx-DsRed mice was immunostained for CC-1, a commonly used marker of mature oligodendrocytes (gray). Neither Venus+ oligodendrocyte progenitor cells (OPCs, green) nor DsRed+ neuroblasts (red) were positive for CC-1. Scale bar: 10 µm.

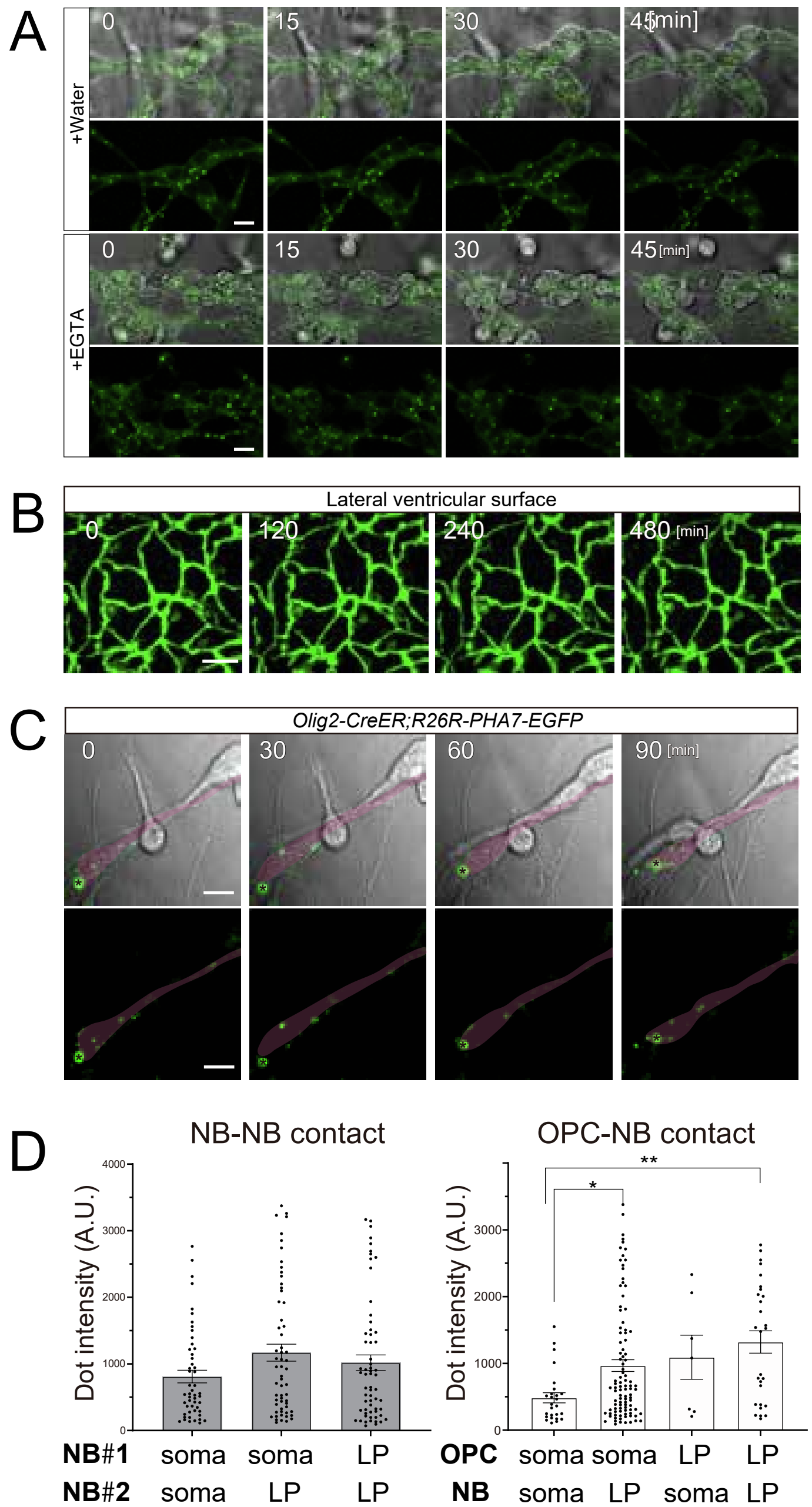

Figure S4. Characterization of PHA7-EGFP signals in V-SVZ explant cultures, related to Figure 5.

(A) Migrating cells in R26R-PHA7-EGFP;Nestin-Cre explant cultures treated with water (control, top) or 5 mM calcium chelator EGTA (bottom) just before imaging. Following EGTA application, the punctate PHA7-EGFP (green) vanished while a single dot per cell (centriole) remained.

(B) Time-lapse imaging of stable, continuous PHA7-EGFP (green) in ventricular surface containing ependymal cells (large, polygonal) and neural stem cells (small) from R26R-PHA7-EGFP;Nestin-Cre explant cultures.

(C) Time-lapse imaging of Olig2-CreER;R26R-PHA7-EGFP explant cultures. PHA7-EGFP (green)-expressing oligodendrocyte progenitor cell (OPC), confirmed to be Olig2-positive by post-hoc immunostaining after live imaging, is highlighted with semi-transparent red overlays. The distribution of EGFP puncta changes dynamically as the OPC migrates alongside unlabeled neuroblasts. Asterisks indicate debris visible in the bright-field channel.

(D) Quantification of intensity of PHA7-EGFP puncta counted in Figure 5I. The intensity at neuroblast–neuroblast (NB–NB, left) and neuroblast–OPC (right) contact sites was measured. Contact types were classified based on the contacting subcellular domain, defined as either the soma or the leading process (LP), as indicated. Each dot represents one punctum. From 2 independent experiments, OPC–NB (34 sites) and NB–NB (41 sites) contacts were analyzed, with dot distributions as follows: OPC–NB—26 soma–soma, 99 soma–process, 7 process–soma, 28 process–process; NB–NB—51 soma–soma, 60 soma–process, 62 process–process. Data are mean ± SEM. *$p < 0.05$, **$p < 0.01$. One-way ANOVA followed by Tukey' s multiple comparisons test. Scale bars: 10 μm: A –C.
